# Unique DNA impacts on transient Expi293^TM^PRO expression for enhancing biotherapeutic protein production

**DOI:** 10.3389/fbioe.2026.1786185

**Published:** 2026-04-14

**Authors:** Bo Hee Shin, Jing Zhou, Audrey M. Vogt, Lauren Gebhardt, Molica Abel, Hamza Sahil, Fabrice Lamour, Anna Kimeu, Ian R. Wallace-Moyer, Sean Lim, Laura Lin, Aaron M. D’Antona, Xiaotian Zhong

**Affiliations:** BioMedicine Design, Preclinical and Translational Sciences, Pfizer Research and Development, Cambridge, MA, United States

**Keywords:** Chinese hamster ovary, empty expression vector, human embryonic kidney 293, mammalian transient gene expression, surface-display molecule, three-snapshot approach, transfected plasmid DNA

## Abstract

**Introduction:**

Mammalian transient gene expression technology is an essential tool for biotherapeutics engineering and production. Various reported transient expression systems in human embryonic kidney (HEK) 293 or Chinese hamster ovary (CHO) cells display different expression patterns and production yields, yet the mechanisms to which these patterns and yields can be attributed remain unknown.

**Methods:**

To identify the determining factors, we have dissected the transient expression processes through a three-snapshot approach for four commercially available transient production systems: Expi293^TM^PRO, Expi293F™, ExpiCHO-S™, and CHO4Tx®. A novel surface-display molecule fused with a CD4 transmembrane domain was designed to utilize flow cytometry to monitor transient expression stages of reaching cell surfaces prior to release into the extracellular medium. A cytosolic green fluorescence protein (GFP) was transfected as a readout for transfection efficiency, DNA transcription, and protein translation. The final stage of transient expression was monitored by expression titers for antibody proteins, which reflected the secretion and accumulation of protein products in culture media.

**Results:**

The data showed that in response to titrated amounts of transfected DNAs for both the surface-display fusion molecule and the cytosolic GFP, Expi293^TM^PRO exhibited a unique sigmoidal-like curve, which was different from the logarithmic-like saturation curves for three other tested cell hosts. These results indicated that the Expi293^TM^PRO cells possessed a high sensitivity to subtle changes in the quantities of transfected DNAs at the stage of protein synthesis. The Expi293^TM^PRO cells were found to achieve very high transient expression, with more than 40 constructs attaining a titer of more than 1 g/L, which could likely be credited to their unusual capability in utilizing transfected DNA for protein synthesis. Interestingly, when the plasmid DNAs encoding the target genes were replaced with the DNAs of empty expression vectors for transfection, a dramatic expression enhancement during the early production phase was observed for the transient Expi293^TM^PRO system.

**Conclusion:**

These results together suggested that the efficiency in handling transfected DNAs for protein synthesis (i.e., the ability to make intracellularly up-taken plasmid DNA complexes competent for DNA transcription in the nucleus) is likely a key limiting factor for effective transient production.

## Introduction

The transfer of exogenous genetic material into desired cells is critical for a number of therapeutic applications, ranging from viral antigen vaccines and siRNA for treating rare inherited diseases (Sharma et al., 2021; Pozzi and Caracciolo, 2023) to recombinant biologics production (Wurm, 2004; Gutierrez-Granados et al., 2018). For biotherapeutics engineering and manufacturing, nonviral deliveries of plasmid DNAs into mammalian cells are important rate-limiting steps for chromosomal or episomal gene expression. Understanding how the exogenic DNA elements harness cell host machinery for efficient protein production has been the subject of intensive research over the past two decades (Pezzoli et al., 2017; Wang and Guo, 2020; Fus-Kujawa et al., 2021; Patel et al., 2021; Oliviero et al., 2022; Bachhav et al., 2023; Kelsall et al., 2023).

Transient gene expression (TGE) technologies in mammalian cells can rapidly generate high-quality protein materials in days due to episomal expression without the need for chromosomal integration, and with relevant post-translational modifications as well as high stringency in protein folding (Pham et al., 2006; Baldi et al., 2007; Geisse, 2009; Jager et al., 2015; Gutierrez-Granados et al., 2018). Human embryonic kidney (HEK) 293 or Chinese hamster ovary (CHO) cells are the two major TGE hosts, owing to their ease of manipulation and broad utilization in clinical manufacturing. During the TGE processes, plasmid DNAs encoding genes of interest (GOI) behind a strong mammalian promoter are typically introduced into mammalian cells through complex formation with transfection reagents or by DNA electroporation (Steger et al., 2015; Gonzalez-Rivera et al., 2024). The common transfection reagents include cationic lipids (Zhou et al., 2023), cationic polymers, and complex blends with DNA-binding proteins (Nagy and Watzele, 2006), which can mask the negative charge of the DNAs and interact with membrane lipids through hydrophobic cores (Kim and Eberwine, 2010; Chong et al., 2021; Fus-Kujawa et al., 2021). Because of the broad utilization for biotherapeutic research, tremendous progress has been made in TGE technologies as a result of two decades of rigorous investigation. A number of high-performance transient systems in HEK293 and CHO have been reported achieving >1 g/L of expression titers for a few protein constructs (Backliwal et al., 2008; Daramola et al., 2014; Rajendra et al., 2015; Steger et al., 2015; Stuible et al., 2018a; Zhong et al., 2019; Schmitt et al., 2020; Gonzalez-Rivera et al., 2024; Wu et al., 2024), making the TGE a competitive production strategy with potential for clinical studies (Stuible et al., 2018b; Bolisetty et al., 2020; Kelley et al., 2022; Rodriguez-Conde et al., 2022).

Despite these breakthroughs, the TGE systems still face substantial unaddressed questions and technological challenges. Many low expressors from screening-campaign-isolated hits or rationally designed constructs cannot be expressed at acceptable levels. More importantly, achieving universally high expression titers comparable to those from stable chromosomal expression remains a distant goal for the TGE systems. The molecular mechanisms by which exogenously delivered plasmid DNAs harness host cell machinery for protein synthesis and secretion are unclear. Significant differences in terms of expression titers and patterns were found among the transient HEK293 and CHO systems. The key factors that dictate these differences are unknown. Specifically, the causes for the delayed transient expression in CHO versus HEK293 are unidentified (Zhong et al., 2019). It remains unknown why the overall expression titers in transient expression are substantially lower than those in stable expression, even though many more expression cassettes and gene copies are delivered into the transient host cells (Carpentier et al., 2007; Cohen et al., 2009). Addressing these critical questions should help advance the understanding of the TGE technologies *per se* and further develop production systems.

In this study, we set out to improve and optimize the TGE systems for therapeutic protein production through a direct comparison of four commercially available transient systems: Expi293F™ (Zhou et al., 2023), ExpiCHO-S^TM^ (Liu et al., 2015; Jain et al., 2017; Zhong et al., 2019), CHO4Tx® (Gebhardt et al., 2025), and the recently launched Expi293^TM^PRO (www.thermofisher.com). A three-snapshot approach was utilized for analyzing the protein production processes in these TGE systems. Transfections of a green fluorescence protein (GFP) could monitor the consequences of plasmid DNA uptake into the nucleus for transcription and cytosolic protein synthesis. The design of a novel surface-display fusion molecule can further examine the outcomes from ER protein translocation and membrane vesicle trafficking to the cell surface. Finally, the expression titers for secreted antibodies could assess protein release and accumulation in the culture medium. The results from the study indicated that Expi293^TM^PRO exhibited a unique pattern in response to the varied amounts of transfected plasmid DNAs, which was different from those in other transient systems. Unexpectedly, when the GOI-encoding plasmid DNAs were replaced with the DNAs of an empty expression vector for transfection, a dramatic expression enhancement during the early production phase was observed for the transient Expi293^TM^PRO system. These data together suggest that the efficiency in turning transfected plasmid DNAs into the intracellular plasmid DNAs competent for transcription and translation might be a key factor for transient protein production.

## Materials and methods

### DNA constructs

DNA fragments encoding a human CD4 transmembrane domain (aa123–185) with a five-glycine linker in between (5′-GGT​GGA​GGC​GGG​GGA​CCA​ATG​GCC​CTG​ATT​GTG​CTG​GGG​GGC​GTC​G-CCG​GCC​TCC​TGC​TTT​TCA​TTG​GGC​TAG​GCA​TCT​TCT​TCT​GTG​TCA​GGT​GCC​GGC​ACC​GAA-GGC​GCC​AAG​CAG​AGC​GGA​TGT​CTC​AGA​TCA​AGA​GAC​TCC​TCA​GTG​AGA​AGA​AGA​CCT​G-CCA​GTG​TCC​TCA​CCG​GTT​TCA​GAA​GAC​ATG​TAG​CCC​CAT​T-3′) in-frame fused to the C-terminus of an antibody heavy chain (target-HC-transmembrane domain (TMD)) was synthesized by PCR amplification using KOD Hot Start DNA Polymerase (Sigma-Aldrich, St. Louis, MO, United States, Cat. #71086-4). Primers were purchased from Integrated DNA Technologies (Coralville, IA, United States). After generating target-HC-TMD, the insert was infused into a pTT5 expression vector by using an In-Fusion® HD Cloning Kit (Takara Bio United States, San Jose, CA, United States, Cat. #102518). Target-HC-TMD vector was validated with restriction enzyme cutting with EcoRI-HF (New England BioLabs, Ipswich, MA, United States, Cat. #R3101S) and BamHI-HF (New England BioLabs, Cat. #R3136L). The final constructs were verified by Nanopore sequencing (RBK 114.96 Library Prep Kit, Oxford Nanopore Technologies, Oxford, United Kingdom).

### Plasmid DNA preparation

Plasmid DNA was transformed into TOP10 Competent Cells (Thermo Fisher Scientific, Waltham, MA, United States, Cat. #C404010). Transformed cells were plated onto LB agar supplemented with carbenicillin (100 ng/μL) and incubated overnight at 37 °C. Individual colonies were selected and inoculated into 60 mL of Thomson Plasmid+® medium (Thomson Instrument Company, Carlsbad, CA, United States, Cat. #NC0085590) and cultured for 24 h at 37 °C with agitation at 225 rpm. Plasmid extraction was performed using the Biotage® PhyPrep system (Biotage®, Uppsala, Sweden) at the maxiprep scale. Plasmid concentration, A260/280, and A260/230 were recorded using the Lunatic quantification system (Unchained Labs, Brighton, MA, United States). All purified plasmids were verified by Nanopore sequencing.

### Mammalian cell culture

Expi293^TM^PRO cells (Cat. #A40001140), Expi293F™ cells (Cat. #A14527), and ExpiCHO-S™ cells (Cat. #A29127) were obtained from Thermo Fisher Scientific, and CHO4Tx® cells were purchased from Magellan Biologics (Novas, Portugal). Cells were cultured in Multitron incubator shakers (INFORS HT, Bottmingen, Switzerland) or Kuhner incubator shakers (Kuhner, Birsfelden, Switzerland) with 8% CO_2_ at 37 °C with high humidity and regularly maintained in Thomson Optimum Growth® shake flasks (Thomson Instrument Company) or Corning Erlenmeyer flasks (Corning Inc., Corning, NY, United States) with Expi293^TM^PRO Expression Medium (Thermo Fisher Scientific, Cat. #A4000135501), Expi293F™ Expression Medium (Thermo Fisher Scientific, Cat. #A1435101), ExpiCHO-S^TM^ Expression Medium (Thermo Fisher Scientific, Cat. #A2910001), and CHO4Tx® Cultivation Medium (CM, Magellan Biologics) containing 4 mM of L-glutamine (L-Gln) (Thermo Fisher Scientific), respectively. Viable cell density and cell viability were monitored by a Vi-CELL^TM^ cell viability analyzer (XR Model, Beckman Colter Life Sciences, Indianapolis, IN, United States).

### DNA titration and transfection

Plasmid DNAs were prepared at 1 mg/L of culture volume as 100% target DNA usage for ExpiCHO-S^TM^, Expi293F™, and Expi293^TM^PRO cells based on the manufacturer’s guidelines. Plasmid DNAs for CHO4Tx® were prepared at 6.5 mg/L of culture volume as 100% target DNA usage according to the manufacturer’s guidelines. At 1 mg/L of culture volume, the empty expression vector pTT5 for Expi293^TM^PRO was at 100% usage. DNA titrations for transfection were calculated accordingly as percentages of the target DNA used.

### Transfection for surface display and GFP

A 135-µL aliquot of Expi293^TM^PRO Transfection Reagent (Thermo Fisher Scientific, Cat. #A40001638B2F) was mixed with target-HC-TMD and light-chain (LC) DNA in 3 mL of cold Opti-Plex™ Complexation Buffer (Thermo Fisher Scientific, Cat. #A4096801) or Opti-MEM^TM^ Reduced Serum Medium (Thermo Fisher Scientific, Cat. #31985070). After 5 min incubation at room temperature (RT), the DNA and transfection reagent complexes were transferred to 30 mL of Expi293^TM^PRO cells with >99% viability at 5 × 10^6^ cells/mL. An 81-µL aliquot of ExpiFectamine™ 293 (Thermo Fisher Scientific, Cat. #100014995) was used for 30 mL of Expi293F™ cells, and a 96-µL aliquot of ExpiFectamine^TM^ CHO (Thermo Fisher Scientific, Cat. #100033022) was used for 30 mL ExpiCHO-S™ cells. Both the DNAs and the transfection reagents were first diluted in Opti-MEM^TM^ prior to the mixing incubation at room temperature (5 min for ExpiFectamine™ 293 and 2.5 min for ExpiFectamine™ CHO). The DNA and ExpiFectamine™ complexes were added to either Expi293F™ cells at 3.8 × 10^6^/mL or ExpiCHO-S^TM^ cells at 6.0 × 10^6^/mL with >99% viability. Expi293^TM^PRO, Expi293F™, and ExpiCHO-S^TM^ cells were incubated in Kuhner shaker incubators or Multitron shaker incubators at 37 °C, 130 rpm, 8% CO_2_ for 20 h. For CHO4Tx® transfection, 1 day before transfection, cells were aliquoted to 30 mL at 2.0–3.0 × 10^6^ cells/mL and pelleted via centrifugation, 3,000 *× g* for 5 min. The cell pellets were resuspended with 30 mL of fresh CHO4Tx® CM and incubated at 37 °C, 100 rpm, 8% CO_2_ for 1 day. On the day of transfection, 30 mL of cells at 6 × 10^6^ cells/mL were pelleted via centrifugation, 3,000 *× g* for 10 min, then resuspended in 15 mL of CHO4Tx® Transfection Medium (TM, Magellan Biologics) and incubated for 3 h at 31 °C, 5% CO_2_. After 3 h, 15 mL of CHO4Tx® Production Medium (PM, Magellan Biologics) was added to the flask. CHO4Tx® was stored in Kuhner shaker incubators or Multitron shaker incubators at 31 °C, 120 rpm, 5% CO_2_ for 20 h. The cells from all the cell lines were harvested and analyzed after 20 h post-transfection. For GFP transfection, GFP plasmid DNA was used instead of target-HC-TMD and LC in the aforementioned transfection condition for each cell line.

### Transient protein production in Expi293^TM^PRO and Expi293F™

A 4.5-µL aliquot of Expi293^TM^PRO Transfection Reagent per 1 mL culture volume was mixed with DNA in cold Opti-Plex™ Complexation Buffer or Opti-MEM^TM^. After 5 min incubation at RT, the DNA and transfection reagent complexes were transferred to Expi293^TM^PRO Cells at 5 × 10^6^ cells/mL. ExpiFectamine™ 293 was used at 2.7 µL per 1 mL of cell culture. DNA and ExpiFectamine™ 293 were first diluted in Opti-MEM^TM^, prior to the mixing incubation at RT for 5 min. The DNA and ExpiFectamine™ complexes were added to Expi293F™ cells at 3.8 × 10^6^/mL. The transfected Expi293F™ was fed with Expi293™ Transfection Enhancer-1 (Thermo Fisher Scientific, Cat. #100013864) and ExpiFectamine™ 293 Transfection Enhancer-2 (Thermo Fisher Scientific, Cat. #A14350-02), while Expi293^TM^PRO were fed with Expi293^TM^ PRO Enhancer (Thermo Fisher Scientific, Cat. #A40000966) and Expi293^TM^ PRO Feed (Thermo Fisher Scientific, Cat. #A40001356-02) after 20 h post-transfection and incubated in incubators at 37 °C, 120 rpm, 8% CO_2_ for 6 days.

### Flow cytometry analysis

At 20 h post-transfection for the plasmid DNA encoding the surface-display molecule, the transfected cells were collected and washed with Dulbecco’s phosphate-buffered saline (DPBS) (Thermo Fisher Scientific, Cat. #14-040-133) and labeled with goat anti-human IgG Fc, PE-conjugated (Thermo Fisher Scientific, Cat. #12-4998-82) at a 1:100 dilution with DPBS. Doublet cells were excluded, and PE % positive cells and mean fluorescence intensity were acquired by a BD LSRFortessa™ cell analyzer (Beckon Dickinson, Franklin Lakes, NJ, United States) with FACSDiva software (Beckon Dickinson). FlowJo v.10 (FlowJo LLC, Ashland, Oregon, United States) was used for data analysis. For the transfection with the plasmid DNAs encoding GFP, the transfected cells were collected and washed with DPBS. Doublet cells were excluded, and FITC-positive cell % and mean fluorescence intensity were acquired by a BD LSRFortessa™ cell analyzer with FACSDiva software. FlowJo v.10 was used for data analysis.

### Octet titer determination

Cells were spun down at 3,500 *× g* for 10 min to separate the cell pellet and the conditioned medium. Antibody titer was detected from the collected conditioned medium by Octet® bio-layer interferometry (BLI)-based ProA Biosensors (Sartorius AG, Göttingen, Germany, Cat. #18-5010) in an Octet red instrument (Sartorius AG).

### ProA capture using the ÄKTA system

Produced antibodies were purified by Protein A capture via 1 mL HiTrap™ MabSelect SuRe (Cytiva, Marlborough, MA, United States, Cat. #29-0491-04) in the ÄKTA Pure3 system (Cytiva). UNICORN software (Cytiva) was used for system control.

### SDS-PAGE analysis

Protein purified by using ProA was loaded on SDS-PAGE under both non-reducing and reducing conditions using a reducing reagent (Thermo Fisher Scientific, Cat. #NP0009) and sample boiling at 95 °C. Intact antibody molecular weight was confirmed by the gel under non-reducing conditions; heavy-chain and light-chain molecular weights were confirmed by the gel under reducing conditions. The gel was stained with AcquaStain Protein Gel Stain (Bulldog-Bio, Portsmouth, NH, United States, Cat. #AS001000).

### Analytical size-exclusion chromatography analysis

The percentages of protein of interest (POI) were analyzed by analytical size-exclusion chromatography (aSEC) using high-performance liquid chromatography (HPLC). Agilent 1200 (Agilent Technologies, Santa Clara, CA, United States) with YMC Pack Diol200 (YMC America, Devens, MA, United States, Cat. #DL20S05-3008WT) was used for sample running, and OpenLab CDS 2 software (Citrix OpenLab, Fort Lauderdale, Florida, United States) was utilized for system control, data acquisition, and analysis.

## Results

### Dissecting transient expression processes through a three-snapshot approach

**FIGURE 1:**
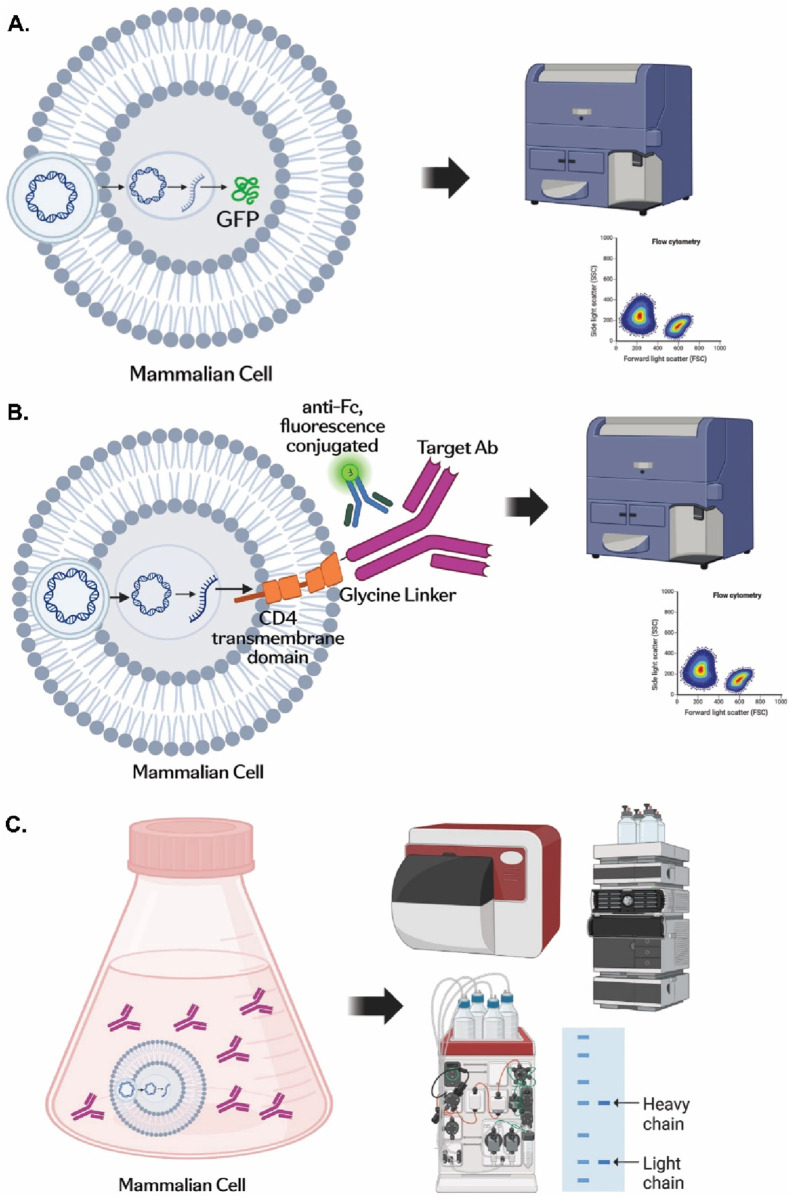
Dissecting the transient expression processes through a three-snapshot approach. **(A)** Plasmid DNAs encoding a cytosolic GFP protein could be transfected into mammalian cells, and the GFP expression could be detected by flow cytometry. The fluorescence signal was detected by LSRFortessa™ Flow Cytometry and analyzed by FlowJo. **(B)** A surface-display molecule was designed by incorporating a CD4 transmembrane domain to the C-terminus of an antibody heavy chain linked with a glycine linker. The surface-display antibody encoded by plasmid DNAs could be expressed outside the plasma membrane of transfected mammalian cells and detected by a fluorescence-conjugated anti-Fc antibody. **(C)** Antibody production titers and product quality could be monitored by ProA-capturing, Octet analysis, aSEC, and SDS-PAGE. BioRender software was used for graphic drawings (www.biorender.com).

First, plasmid DNAs encoding a cytosolic GFP protein were utilized as a readout for transfection efficiency and cytosolic protein expression through flow cytometry analysis (Figure 1A). Second, as shown in Figure 1B, a surface-display antibody molecule was constructed in which an antibody heavy chain (HC) was fused with a C-terminal transmembrane domain (TMD) of human CD4 protein through a five-glycine linker, as described in Materials and Methods. This design could allow the detection of the surface expression of the resulting molecule by flow cytometry analysis with fluorescence-conjugated anti-Fc antibody. The flow cytometry analysis could be performed 20 h post-transfection on the transfected cells. The high sensitivity of flow cytometry analysis enabled us to study the early stages of protein production processes during which the amount of secreted antibody proteins is typically too low to be detected in the culture medium. As a final snapshot on the TGE processes shown in Figure 1C, expression titers and product quality of antibody proteins secreted into the culture medium could be monitored by ÄKTA-based protein A (ProA) capturing, Octet BLI-based ProA biosensor analysis, HPLC-based aSEC analysis, and SDS-PAGE.

### GFP protein expression in Expi293^TM^PRO exhibited a unique sigmoidal-like curve in response to the DNA amounts, unlike the curves of Expi293F™, ExpiCHO-S™, and CHO4Tx®

The four commercially available transient systems in the market include two HEK293-based TGE systems (Expi293F™ and Expi293^TM^PRO) and two CHO-based TGE systems (ExpiCHO-S™ and CHO4Tx®). To understand how the expression of a cytosolic protein behaved in response to the changes in the quantities of transfected DNAs, the plasmid DNAs encoding cytosolic GFP were transfected into the four cell hosts with different amounts of plasmid DNA titrated. The cell viabilities for all transfected cells were high (96%–99%) and were not affected by the amounts of transfected DNA. The transfected cells were analyzed by flow cytometry, with percentages of fluorescence-positive cells and mean fluorescence intensity (MFI) measured by LSRFortessa™ flow cytometry.

**FIGURE 2:**
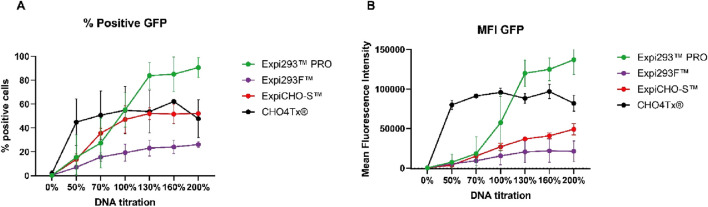
GFP protein expression in Expi293^TM^PRO exhibited a unique sigmoidal-like curve in response to the DNA amounts, different from the responses of Expi293F^TM^, ExpiCHO-S™, and CHO4Tx®. The plasmid DNA encoding GFP was transfected into four cell lines with a titrated amount, as described in Materials and Methods. At 20 h post-transfection, transfected cells were collected. Percentages of positive transfected cell populations and mean fluorescence intensity were measured by LSRFortessa™ flow cytometry. **(A)** Percentage of positive transfected cell populations (n = 2–4 ± S.D.). **(B)** Mean fluorescence intensity of transfected cells (n = 2–4 ± S.D.).

As shown in Figure 2, cytosolic GFP protein exhibited different patterns for percentages of positive transfected cell populations and MFIs among Expi293F™, Expi293^TM^PRO, ExpiCHO-S™, and CHO4Tx® cells. In Figure 2A, the percentages of positive cells for Expi293F™ and ExpiCHO-S™ gradually increased as the DNA amounts increased, while that for CHO4Tx® reached a plateau even at 50% of the target DNA usage. ExpiCHO-S™ (∼50% of total cells) and CHO4Tx® (∼60% of total cells) reached the max level at the amount of 100% of target DNA usage, whereas Expi293F™ (∼25% of total cells) saturated at approximately 130% of target DNA usage. In contrast, Expi293^TM^PRO possessed the highest percentage of positive transfected cells (∼90% of total cells) at the amount of 130% of target DNA usage. It showed a sharp, fourfold increase from 50% to 130% of target DNA usage.

Figure 2B shows the MFI of Figure 2A for total GFP protein expression. ExpiCHO-S™ and Expi293F™ had a saturation expression at 130% of target DNA usage, whereas CHO4Tx® saturated at 50%. Interestingly, Expi293^TM^PRO exhibited a unique sigmoidal-like curve in response to the transfected DNA amounts. It showed a sharp, sixfold increase as the targeted DNA usage rose from 70% to 130%, and its MFI continued to increase up to 200%. Expi293^TM^PRO not only exhibited high transfection efficiency but also significantly higher expression density for the cytosolic GFP than did Expi293F™, ExpiCHO-S™, and CHO4Tx®. Because GFP is a cytosolic protein, the biological events that caused the differences in the DNA response curves between Expi293^TM^PRO and other cell hosts appeared to take place during the protein synthesis stage.

### Flow cytometry analysis revealed that Expi293^TM^PRO also exhibited a unique sigmoidal-like curve in response to the transfected DNA amounts for the surface-display fusion molecule, unlike the logarithmic-like saturation curves for Expi293F™, ExpiCHO-S™, and CHO4Tx®

**FIGURE 3:**
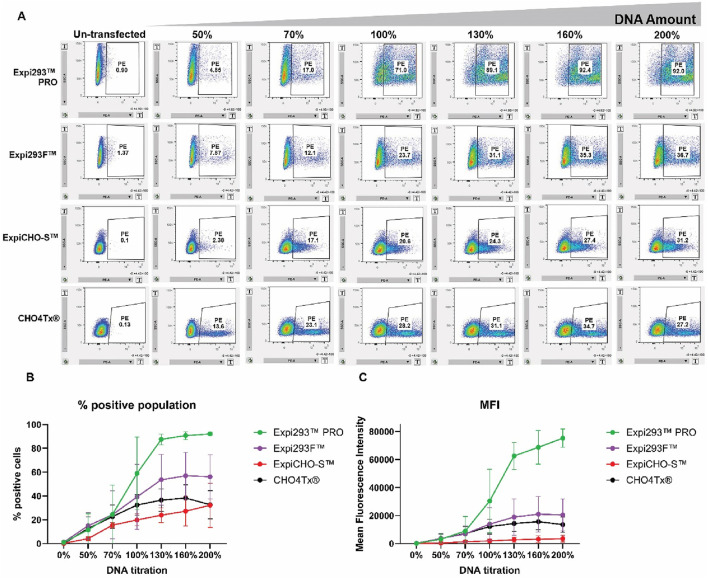
Flow cytometry analysis revealed that Expi293^TM^PRO exhibited a unique sigmoidal-like curve in response to the DNA amounts for the surface-display fusion molecule, different from the logarithmic-like saturation curves for Expi293F^TM^, ExpiCHO-S™, and CHO4Tx®. Various amounts of plasmid DNAs were titrated for the transfection into four different cell hosts, as described in Materials and Methods. Cells were collected and labeled with PE-conjugated anti-IgG Fc 20 h post-transfection. Percentages of PE-positive transfected cell populations and mean fluorescence intensity were measured from LSRFortessa™ flow cytometry. **(A)** Representative scatter plot images for transfected cells. **(B)** Percentage of positive transfected cell populations (n = 3 ± S.D.). **(C)** Mean fluorescence intensity of transfected cells (n = 3 ± S.D.).

To compare the transient expression efficiency during the early stages of protein production processes for the four commercially available transient systems, the plasmid DNAs encoding the surface-display antibody were transfected as described in Materials and Methods, with different amounts of plasmid DNAs being titrated. The antibody HC:LC DNA ratios were fixed at 1:1. The cell viabilities for all transfected cells were 96%–99%. The transfected cells were then analyzed by flow cytometry for surface expression at 20 h post-transfection. The transfected cells were collected and labeled with an anti-IgG Fc-PE (phycoerythrin) conjugated antibody. Percentages of fluorescence-positive cells and MFI were measured by LSRFortessa™ flow cytometry (Figure 3A).

As shown in Figures 3A,B, Expi293F™, Expi293^TM^PRO, ExpiCHO-S™, and CHO4Tx® also exhibited different percentages of positive transfected cell populations. The percentages of positive cell population for Expi293F™, ExpiCHO-S™, and CHO4Tx® gradually increased as the DNA amounts increased (Figure 3B). ExpiCHO-S™ (∼20% of total cells) and CHO4Tx® (∼30% of total cells) started leveling off at 70% and 100% target DNA usage, respectively, whereas Expi293F™ (∼50% of total cells) saturated at approximately 130% target DNA usage. In contrast, Expi293^TM^PRO possessed the highest percentage of positive transfected cells (∼90% of total cells). It again showed a sharp increase in positive cell population percentage from 50% target DNA usage to 130% and reached the plateau at 130%.

Figure 3C showed the MFI of Figure 3A, which was the reflection of the total surface expression of the surface-display antibody molecule. Consistent with our previous report that ExpiCHO-S™ had a delayed protein expression (Zhong et al., 2019), MFI for ExpiCHO-S™ was very low at 20 h post-transfection. Both Expi293F™ and CHO4Tx® displayed a logarithmic-like saturation curve in response to the titrated amounts of transfected DNAs. Interestingly, Expi293^TM^PRO also exhibited a unique sigmoidal-like DNA response curve. It showed a sharp increase in MFI from the amount of 70% target DNA usage to that of 130%, and its MFI continued to increase up to 200% target DNA usage. These results together indicated that Expi293^TM^PRO appeared to be more sensitive to changes in DNA amounts than other cell hosts.

### A new production protocol for Expi293^TM^PRO was established according to the DNA responses and was found to dramatically increase transient expression titers

**FIGURE 4:**
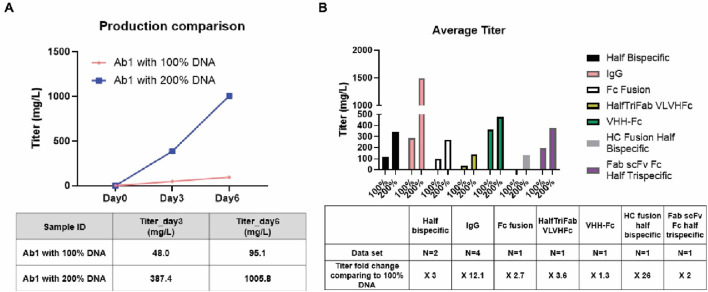
A new transfection protocol for Expi293^TM^PRO established according to the DNA response curve was found to dramatically increase transient expression titers. Following the procedure described in Materials and Methods, 100% and 200% of the target DNA were used for transfection in Expi293^TM^PRO cells. **(A)** Production comparison for Antibody-1 (Ab-1) between the 100% target DNA usage protocol and the 200% target DNA usage protocol. **(B)** Production comparison among different scaffolds between the 100% target DNA usage protocol and the 200% target DNA usage protocol.

Based on the findings shown in Figures 2, 3, antibody-1 (Ab-1) was expressed in Expi293^TM^PRO with 100% and 200% of standard DNA used. As shown in Figure 4A, the 100% target DNA usage gave an expression titer of 96.1 mg/L, while the 200% target DNA usage dramatically improved the transient expression titer by more than 10-fold (1,005.8 mg/L). To further confirm the advantage of the 200% target DNA usage, a number of protein scaffolds have been tested, including regular IgG, bispecific antibody halfmers (half bispecifics), fragment antigen-binding (Fab) light-chain variable domain (VL) HC-variable-domain (VH)-Fc fusion (HalfTriFabVLVHFc), single-domain nanobody-Fc fusion (VHH-Fc), HC-fusion halfmer of bispecific (HC fusion half bispecific), and Fab single-chain variable fragment (scFv)-Fc fusion (Fab-scFv-Fc half trispecific). As shown in Figure 4B, the 200% target DNA usage protocol was found to exhibit significant titer improvements across these scaffold formats. Notably, the regular IgG antibodies had the highest expression improvement.

### A broad utilization of the new Expi293^TM^PRO protocol in the biotherapeutic portfolio resulted in achieving expression titers of >1 g/L for 40 constructs

**FIGURE 5:**
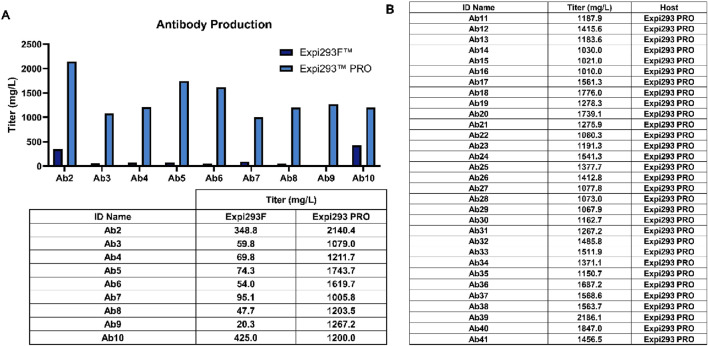
A broad utilization of the new Expi293^TM^PRO protocol in a biotherapeutic portfolio achieved titers of >1 g/L for 40 constructs. A. Titer comparison between Expi293^TM^PRO and Expi293F™. Tables from both **(A,B)** showed the antibody constructs reaching titers of more than 1 g/L.

As shown in Figure 5A, the production levels in Expi293^TM^PRO were more than 10-fold higher than those in Expi293F™. When the new Expi293^TM^PRO protocol was utilized for broader therapeutics production, significant expression enhancements compared to those in Expi293F™ have been observed. Outstandingly, 41 antibody constructs with unique sequences were able to achieve titers of >1 g/L (Figures 5A,B). This group of higher expressors represented approximately 30% of the total target antibody proteins tested in this study. These results confirm that implementing a new transfection protocol significantly improves production titers in Expi293^TM^PRO cells.

### When the GOI-encoded plasmid DNAs were replaced with the DNAs of an empty expression vector for transfection, a significant expression enhancement was detected for low expressors

It has been previously reported that the GOI-encoded plasmid DNAs can be replaced with non-specific filler DNAs without decreasing expression titers (Kichler et al., 2005; Pradhan and Gadgil, 2012; Rajendra et al., 2012; Greene et al., 2021). To investigate the effect of replacement with empty expression vector DNA on reducing DNA usage in the newly established 200% target DNA protocol, the plasmid DNAs encoding the surface-display antibody were replaced with 50% of empty expression vector DNA pTT5.

**FIGURE 6:**
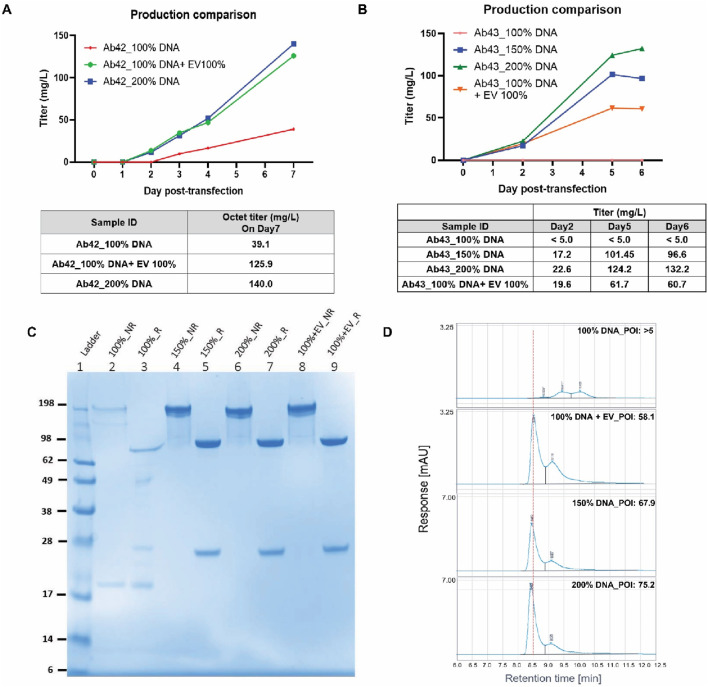
When the GOI-encoded plasmid DNAs were replaced with the DNAs of an empty expression vector for transfection, a significant expression enhancement was detected. Hundred percent of target DNA usage, 200% of target DNA usage, or 100% target DNA plus 100% empty vector pTT5 DNA (EV) were transfected into Expi293^TM^PRO cells, as described in Materials and Methods. **(A)** Production time course for antibody-42 (Ab-42). **(B)** Production time course for antibody-43 (Ab-43). **(C)** SDS-PAGE analysis on the ProA-purified samples from panel **(B)**. R, reduced condition; NR, non-reduced condition. **(D)** aSEC data on the ProA-purified samples from panel **(C)**.

As shown in Figure 6A, 100% of plasmid DNA encoding antibody-42 plus 100% pTT5 vector achieved titers as high as that of 200% DNA usage, whereas it obtained a much higher titer with only 100% DNA. Similar data were obtained with the antibody-43 construct, in which very little expression with multiple cell hosts was obtained initially (Figure 6B). The new 200% DNA usage protocol increased antibody-43 expression by nearly 20-fold, whereas the titer with 100% pTT5 substitution was increased by nearly 10-fold. Interestingly, because of the higher titers, the protease degradation event that occurred in the 100% DNA usage condition of this construct was also minimized (Figure 6C). One possible explanation for this observation is that the purity of the ProA-captured pool from the low-titer culture sample was significantly lower, leading to the possibility that some host cell proteins may look like cleaved product or that a higher level of residual proteases could cleave the target protein during storage. Consistently, the aSEC profiles were also dramatically improved (Figure 6D). Note that for high-expressor Antibody-2 (Ab-2, >2 g/L with 200% DNA usage, Figure 5A), 100% of plasmid DNA encoding Ab-2 plus 100% pTT5 vector achieved the same expression titer as 100% DNA usage (∼0.6 g/L). Empty vector pTT5 DNAs appeared to have no impact on improving titers for high expressors.

### Expression enhancement by the empty expression vector DNA occurred during the early production phase

**FIGURE 7:**
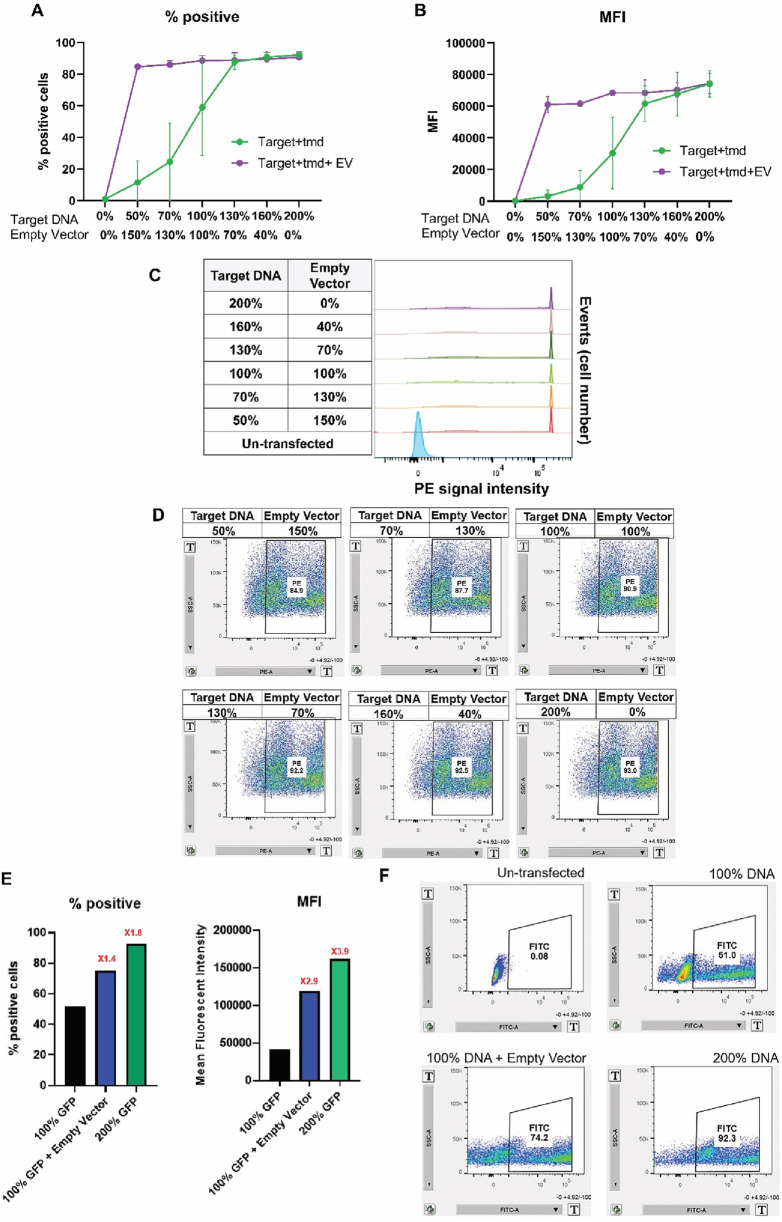
The enhancement effect by empty vector DNA for transfection occurred at the early production phase. Titrated target DNAs (encoding surface-display molecule or GFP) combined with empty vector DNA pTT5 to make up 200% total DNA usage were transfected into Expi293^TM^PRO cells, as described in Materials and Methods. Flow cytometry analysis was performed at 20 h post-transfection. **(A)** Percentage of positive cell populations transfected with plasmid DNA encoding surface-display molecules. **(B)** MFI of transfected cells. **(C)** Histogram profile of Expi293 PRO cells with the indicated DNA amounts. **(D)** Scatter plot images for transfected cells as indicated. **(E)** Percentage of positive cell populations transfected with plasmid DNAs encoding GFP and MFI of the corresponding transfected cells. **(F)** Scatter plot image for the GFP-transfected cells are shown in panel E.

To ascertain a potential mechanism for the effect of empty expression vector, the plasmid DNAs encoding the surface-display molecule were titrated and transfected into Expi293^TM^PRO and compared to the same DNA condition with the co-transfection of empty expression vector pTT5. Various amounts of empty expression vector pTT5 DNA were combined with the corresponding amounts of the GOI-encoding plasmid DNAs to fulfill the final 200% target DNA usage. Flow cytometry with anti-IgG Fc was performed at 20 h post-transfection. Figure 7 shows the percentages of fluorescence-positive cells, MFI, scatter plot profiles, and histogram data. Intriguingly, a dramatic increase in the percentage of fluorescence-positive cells (Figures 7A,D) and MFI (Figures 7B,D) was observed with the supplement of pTT5 DNA. The scatter plots (Figure 7C) indicate that most of the transfected cell populations were shifted from left to right, representing fluorescence positivity. Histogram data (Figure 7D) also show that the empty-expression-vector-added transfection had very high fluorescence density. Regardless of the target DNA amounts, each GOI-encoding plasmid DNA titrated with empty vector showed a similar profile to that of the 200% GOI-encoding plasmid DNA-only transfection, both in terms of the percentages of fluorescence-positive cell population (Figure 7A) and fluorescence intensity (Figure 7B). Similarly, an expression enhancement with empty expression vector DNAs was also detected for cytosolic GFP (Figures 7E,F). When the plasmid DNA encoding GFP was supplemented with the empty vector pTT5 DNA, an increase in percentages of fluorescence-positive cells and the corresponding MFI compared to those from the 100% target DNA usage was also observed (Figure 7E). These results together suggested that the expression enhancement effect with empty vector DNA was likely due to the impact on protein synthesis.

## Discussion

Through dissecting mammalian TGE processes via a three-snapshot approach, this study has revealed some interesting scientific insights into mammalian transient cell hosts. Among the four commercially available cell lines tested, Expi293^TM^PRO cells were found to possess a sigmoidal-like DNA response curve, different from the logarithmic-like curves for Expi293F™, ExpiCHO-S™, and CHO4Tx® cells. Based on the finding that Expi293^TM^PRO cells were sensitive to subtle DNA changes, the investigation has implemented a robust new transfection protocol for high-titer expression to accommodate increasingly complex biotherapeutics engineering. More than 40 therapeutic constructs were found to achieve titers greater than 1 g/L, attaining more frequent stable-production-comparable expression. The study also uncovered an expression enhancement effect by empty vector DNA on Expi293^TM^PRO cells, which was found to take place at the early stages of the transient production process. These findings have revealed not only interesting features about Expi293^TM^PRO cells but also a new hypothesis that the ability to efficiently turn transfected plasmid DNAs into the intracellular plasmid DNAs competent for transcription and translation might likely be a key limiting factor for effective transient production.

Transfection reagents, transfected plasmid DNAs, and their ratios are three important factors for transient transfection processes. In this study, the amount of transfection reagents was held constant at the optimal amounts recommended for each cell host. This raises the possibility that the differing outcomes among the tested cell hosts could also be in part attributed to a higher capacity with the transfection reagent for the Expi293^TM^PRO because its higher titers correlated with its lower ratios of transfection reagent:DNA. The compositions of transfection reagents in the tested commercial systems are not disclosed by the manufacturers, although they are known to be cationic lipids and polymers. Consistent with the commercial protocols, there is an optimal range for the amounts of transfection reagents independent of the DNA ratios. This is likely due to a subtle balance between the DNA uptake efficiency and the toxicity of transfection reagents caused by endosome organelle disruption, presumably through a proton sponge effect in acidic pHs (Wiethoff and Middaugh, 2003). The transfection reagents of Expi293^TM^PRO and Expi293F are interchangeable. Thus, the observed performance differences in each cell host, which were compared with those at the recommended DNA usages (100% usage), are likely attributed to the superior DNA handling capability of the Expi293^TM^PRO cell host.

**FIGURE 8:**
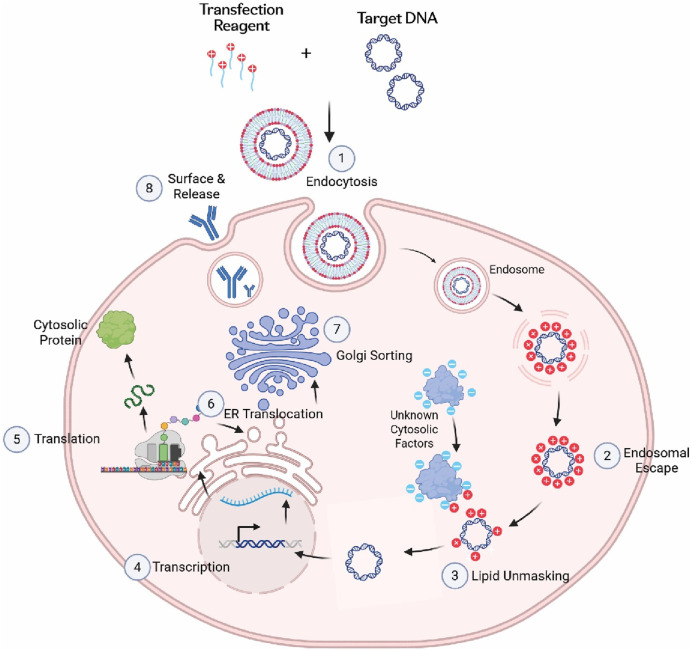
A proposed model for the Expi293^TM^PRO production mechanism. For an antibody protein or a cytosolic protein to be produced by transient Expi293^TM^PRO, a complicated protein production process must take place in mammalian host cells. Eight critical steps are listed in the drawing (www.biorender.com).

A mechanistic model for Expi293^TM^PRO high transient production has been proposed in Figure 8. For an antibody protein to be produced by a TGE system, a complicated protein production process must take place in mammalian host cells. This includes the uptake of plasmid DNA complexes through endocytosis, endosome escape of the DNA complexes, DNA unmasking from transfection reagents, DNA import into nucleus, mRNA transcription from plasmid DNA, mRNA splicing, mRNA export into cytosol, protein translation, ER protein translocation across ER membrane into lumen, protein refolding and post-translational modifications (PTM), ER exit after quality control, Golgi sorting, and further PTM processing, membrane vesicle trafficking to cell surface, and protein release into culture medium. The transfection of plasmid DNAs encoding the cytosolic GFP can be a readout reflecting the combined final outcomes of the uptake of plasmid DNA complexes, the endosome escape of the DNA complexes, DNA unloaded from transfection reagents, DNA import into the nucleus, mRNA transcription from plasmid DNA, mRNA splicing, mRNA export into the cytosol, and protein translation. The transfection of plasmid DNAs encoding the novel surface-display fusion molecule can further examine the outcomes from ER protein translocation across the ER membrane, protein refolding in the ER lumen, Golgi sorting, and vesicle trafficking to the cell surface. The data from this study indicate that the host cell machinery inside Expi293^TM^PRO can efficiently utilize transfected DNAs for proficient protein synthesis. With larger amounts of transfected DNAs, Expi293^TM^PRO cells generated higher expression titers. In contrast, other cell hosts tested in this study showed no further expression increase when increasing the amount of DNA beyond the standard DNA usage. Although several mechanistic improvements could contribute to the superior performance of the Expi293^TM^PRO system, one of our leading hypotheses is that Expi293^TM^PRO could unmask transfection reagents more effectively from the DNA complexes by some unknown cytosolic factors (Figure 8, step 3), generating more transcription-competent DNAs for protein synthesis (Wiethoff and Middaugh, 2003).

One interesting finding from this study is the enhancement effect of empty expression vector DNAs on transient expression. In Figures 6A,B, we compared the titers of the conditions of 100% target DNA usage to those of the conditions of 100% target DNA usage plus 100% empty vector pTT5 DNA. Adding only the backbone DNAs should not increase the mRNA transcripts from the target gene inserts, but the protein titers for low expressors (Ab-42 and Ab-43) were enhanced substantially. This observation is different from the previously reported effect of filler DNAs such as salmon sperm DNA, which can replace target DNAs without decreasing expression titers (Kichler et al., 2005; Pradhan and Gadgil, 2012; Rajendra et al., 2012; Halff et al., 2014; Greene et al., 2021). The empty vector pTT5 utilized in this study is the expression vector for the antibody constructs. In our hands, salmon sperm DNAs did not work well in the production systems we tested. The discrepancy could be because these commercial systems utilize highly productive hosts that do not respond well to filler DNAs. It is also possible that the effect of filler DNA is construct-dependent, as the germline sequences from our antibody constructs are different from those in previous reports. Consistently, even in this study, we found that empty vector DNA pTT5 had good impacts on the low expressors (e.g., Ab42 and Ab43 in Figures 6A,B) but not on some high expressors (e.g., Ab-2). The effects of the empty vector or filler DNAs could both be construct-dependent. It has been shown that the sizes of the DNA and lipid complexes increased as the amount of DNA increased (Greene et al., 2021). This could likely explain the beneficial effects of the filler DNAs because the DNA uptake efficiency is maintained. It is known that cell hosts like HEK293 and CHO have their own preferred uptake sizes for the DNA complexes (Zhou et al., 2023). It has also been reported that sometimes, a smaller amount of plasmid DNA could generate a higher expression titer during TGE (Carreno et al., 2024). Our finding is consistent with an early report stating that an expression enhancement of 40% was observed when not all but one empty vector pLMP was co-transfected in HEK293 cells (Nejepinska et al., 2012).

Several potential mechanisms could explain the impacts of transfected DNAs on Expi293^TM^PRO. A deep sequencing study has revealed that complex random transcription could take place from transiently transfected plasmids in mammalian cells (Nejepinska et al., 2012). Significant amounts of RNA transcripts were found from the regions that were not behind a mammalian promoter. These random DNA regions could have weak promoter activities. When the DNA-dependent polymerases are abundant, these DNA areas could initiate transcription and potentially generate “cryptic anti-sense RNA” to pair with the mRNA transcripts of GOI to form double-stranded (ds) RNAs. Therefore, low expression titer of certain antibodies could be attributed to gene expression suppression of the GOIs in a PKR (dsRNA-dependent protein kinase) and dsRNA-dependent manner (Terenzi et al., 1999), as dsRNA can activate PKR to block protein synthesis or RNase L for mRNA degradation. Adding pTT5 with a strong CMV promoter could result in high-affinity protein-binding of DNA-dependent RNA polymerases to the CMV region; therefore, fewer DNA-dependent RNA polymerases are available for those regions with weak promoter activities. Without the insertion of GOI, the pTT5 vector might produce a short RNA transcript, which is not long enough to increase the chances for pairing with anti-sense cryptic RNA. Therefore, fewer dsRNAs could be produced, and less dsRNA-dependent gene suppression could be detected. The expression titer of the GOI could therefore be enhanced. Another possible mechanism is that futile transcription in the empty expression vector DNAs could slow the active transcription in the GOI-encoding plasmid DNA. With fewer mRNAs produced, the traffic jam caused by the outburst of protein synthesis during transient expression could be relieved, in turn facilitating the protein production process. Reducing transfected DNAs could also result in fewer transcription-competent DNAs and, consequently, fewer mRNAs. However, this modulation affects multiple levels of transient expression processes, including DNA import into the cytosol, DNA import into the nucleus, and DNA transcription. The Expi293^TM^PRO system responds dramatically to the subtle decrease of transfected DNAs, implying a relatively large response range to impacting mRNA amounts. Empty vector supplementation might regulate mRNA levels more delicately. The “traffic jam” effect might only work within a certain range of protein synthesis outburst. Future studies should quantify transgene RNA levels after transfection as the first step to clarify the mechanisms. Further investigation into the DNA impacts on transient Expi293^TM^PRO and other cell hosts is warranted.

High-titer transient expression not only provides rapid protein support for biotherapeutic engineering but also generates predictions for the downstream clinical stable CHO productions. It has been shown that the productivity and quality of a single-chain bispecific T-cell engager produced by stable CHO cell clones can be predicted by transient expression in HEK cells (Diepenbruck et al., 2013). Uncovering and understanding the molecular mechanisms for the TGE systems should make this technology more applicable to biotherapeutics discovery and early development.

## Data Availability

The original contributions presented in the study are included in the article/Supplementary Material; further inquiries can be directed to the corresponding authors.
